# Exploration of Features of Mobile Applications for Medication Adherence in Asia: Narrative Review

**DOI:** 10.2196/60787

**Published:** 2024-11-08

**Authors:** Tzu Wang, Yen-Ming Huang, Hsun-Yu Chan

**Affiliations:** 1 School of Pharmacy College of Medicine National Taiwan University Taiepi City Taiwan; 2 Graduate Institute of Clinical Pharmacy College of Medicine National Taiwan University Taipei City Taiwan; 3 Department of Pharmacy National Taiwan University Hospital Taipei City Taiwan; 4 Department of Industrial Education National Taiwan Normal University Taiepi City Taiwan

**Keywords:** Asia, adherence, application, feature, medication, mobile

## Abstract

**Background:**

Medication is crucial for managing chronic diseases, yet adherence rates are often suboptimal. With advanced integration of IT and mobile internet into health care, mobile apps present a substantial opportunity for improving adherence by incorporating personalized educational, behavioral, and organizational strategies. However, determining the most effective features and functionalities for these apps within the specific health care context in Asia remains a challenge.

**Objective:**

We aimed to review the existing literature, focusing on Asian countries, to identify the optimal features of mobile apps that can effectively enhance medication adherence within the unique context of Asian societies.

**Methods:**

We conducted a narrative review with the SPIDER (sample, phenomenon of interest, design, evaluation, research type) tool. We identified studies on mobile apps for medication adherence from January 2019 to August 2024 on PubMed and Scopus. Key search terms included “Asia,” “chronic disease,” “app,” “application,” “survey,” “experiment,” “questionnaire,” “group,” “medical adherence,” “medication adherence,” “case-control,” “cohort study,” “randomized controlled trial,” “clinical trial,” “observational study,” “qualitative research,” “mixed methods,” and “analysis,” combined using logical operators “OR” and “AND.” The features of mobile apps identified in the studies were evaluated, compared, and summarized based on their disease focuses, developers, target users, features, usability, and use.

**Results:**

The study identified 14 mobile apps designed to enhance medication adherence. Of these, 11 were developed by research teams, while 3 were created by commercial companies or hospitals. All the apps incorporated multiple features to support adherence, with reminders being the most common, present in 11 apps. Patient community forums were the least common, appearing in only 1 app. In total, 6 apps provided lifestyle modification functions, offering dietary and exercise recommendations, generating individualized plans, and monitoring progress. In addition, 6 apps featured health data recording and monitoring functions, with 4 allowing users to export and share records with researchers or health care professionals. Many apps included communication features, with 10 enabling feedback from researchers or health care professionals and 7 offering web-based consultation services. Educational content was available in 8 apps, and 7 used motivation strategies to encourage adherence. Six studies showed that mobile apps improved clinical outcomes, such as blood glucose, lipid, and pressure, while reducing adverse events and boosting physical activities. Twelve studies noted positive humanistic effects, including better medication adherence, quality of life, and user satisfaction.

**Conclusions:**

This review has identified key components integrated into mobile apps to support medication adherence. However, the lack of government and corporate involvement in their development limits the generalizability of any individual app. Beyond basic reminder functions, features such as multiuser support, feedback mechanisms, web-based consultations, motivational tools, and socialization features hold significant promise for improving medication adherence. Further pragmatic research is necessary to validate the effectiveness of these selected apps in enhancing adherence.

## Introduction

Medication adherence refers to how consistently individuals follow their prescribed medication regimen as advised by health care professionals [1]. While medication is powerful for the management of chronic diseases, the average adherence rate stands at 50% worldwide [2]. A meta-analysis revealed that the prevalence of nonadherence among patients taking antihypertensive medications in Asia is 48% [3]. Suboptimal medication adherence is associated with a range of negative consequences, including reduced treatment outcomes, unfavorable disease prognosis, diminished quality of life, worse complications, and frequent hospitalization [4]. Research suggests that patients decide to take medications based on perceived necessity, effectiveness, and safety, and maintain adherence through accessibility, routine, and memory [5]. Both intentional and unintentional nonadherence may occur, where patients may miss doses accidentally or deliberately alter their regimen without counseling their health care providers [6]. Such behaviors contribute significantly to medication-related hospitalizations and preventable deaths, accounting for US $500 billion (16%) of the entire US health care expenditure annually [7].

Efforts to enhance medication adherence involve strategies across behavioral, educational, and organizational domains [8]. Behavioral interventions focus on adapting patients’ surroundings to promote medication adherence, using tools like multidose packs and mapping medication intake to routine [5]. Educational approaches stress the significance of medication in disease management, equipping patients with comprehensive knowledge to empower informed decision-making about their health [9]. Organizational methods streamline medication management through initiatives such as medication synchronization and team-based care, aiming to improve access to medication refill and enhanced adherence [10]. Driven by the advanced integration of IT and mobile internet in health care practice, mobile apps have emerged as a promising avenue for improving adherence by incorporating these strategies and tailoring them to individual needs [11]. Accessible through platforms such as the Apple App Store and Google Play Store, these apps may include reminders, educational resources, and organizational features to support medication adherence. Given the widespread ownership of mobile phones and the tendency of individuals to frequently check their devices, mobile apps offer a convenient and pervasive solution for medication adherence support [12]. Furthermore, they hold substantial potential to enhance patient care and reduce health care costs, particularly considering the significant portion of the population that requires reminders to adhere to their medication regimen [11].

Amidst the onset of the COVID-19 pandemic, digital health interventions such as telehealth and application-based health care emerged in response to the limitations of in-person interventions [13]. The pandemic significantly disrupted the global health system, leading to constrained access to health care services due to surging patient numbers and staff shortages, particularly affecting individuals with chronic diseases who require extensive medical attention [14]. With over half of the global population, approximately 4.3 billion people, now owning smartphones and two-thirds worldwide using the internet, according to the Groupe Speciale Mobile Association’s State of Mobile Internet Connectivity Report 2023, the prevalence of digital connectivity has soared [15]. Research indicates that more than half of mobile phone users have downloaded health-related mobile apps, and about 7 in 10 individuals with such apps used them to track progress on health-related goals in 2020 [16]. Consequently, digital health interventions for chronic disease management became imperative and have demonstrated efficacy in various studies [17,18].

In 2018, Ahmed et al [19] discovered 420 adherence apps available on the Apple App Store and the Google Play Store. Their findings suggest that mobile apps could serve as a potential strategy to tackle medication nonadherence. Haase et al [10] reviewed 30 apps focused on medication adherence and selected 5 with the most ideal features based on the Application Score Card. However, these evaluations overlooked the diverse contexts of health care systems, which are pivotal factors influencing patients’ medication refill and intake behaviors. Asian and western countries demonstrate notable distinctions in their health care insurance systems and cultural perspectives on medication. For instance, Japan, South Korea, and Taiwan operate universal social health insurance systems that cover all citizens and residents [20]. In contrast, the United States primarily relies on private insurance systems, leading to disparities in access to medication [21]. In East Asian countries (eg, Japan, South Korea, and Taiwan), patients can easily obtain medical services and medications at lower costs compared with patients in the United States, who often encounter high out-of-pocket expenses for medications due to the expensive nature of health care [21,22]. In addition, patients with an Asian cultural background tend to hold more negative perceptions about medication. They often view medications as inherently harmful or addictive substances to be avoided [23,24]. Consequently, patients in Asian countries may face fewer barriers to filling prescriptions and refills but may harbor more doubts regarding medication intake. These cultural and systematic nuances should be considered when designing interventions or recommending adherence apps to ensure their effectiveness across diverse patient populations [25].

Over the past few years, there has been a surge in the development of apps targeting medication adherence improvement. However, determining the most effective features and functionalities for these apps remains uncertain. This study was to review the existing literature, with a particular focus on the Asian countries, to identify the optimal features of mobile apps that can effectively enhance medication adherence within the unique context of Asian societies.

## Methods

We used the SPIDER (sample, phenomenon of interest, design, evaluation, research type) framework to craft the research question [26]. So, the research question was “What are the optimal features of mobile apps that are used to improve medication adherence in health care practice in Asian countries?” We followed the principles of the scale for the quality assessment of narrative review articles in conducting this narrative review [27]. Using a qualitative approach, we compared and summarized the features of the selected apps. This involved using an app quality assessment tool, developing a search strategy, applying predefined inclusion criteria for screening apps, comparing and selecting apps, and extracting relevant data.

### Search Strategy

We conducted a literature search on 2 major electronic databases, PubMed and Scopus, to identify studies on the use of mobile apps for medication adherence. The SPIDER framework was used to structure the research question and guide the literature search (Multimedia Appendix 1). The key search terms included “Asia,” “chronic disease,” “app,” “application,” “survey,” “experiment,” “questionnaire,” “group,” “medical adherence,” “medication adherence,” “case-control,” “cohort study,” “randomized controlled trial,” “clinical trial,” “observational study,” “qualitative research,” “mixed methods,” and “analysis,” within the timeframe from January 2019 to August 2024. These terms were combined using logical operators “OR” and “AND” and were restricted to searches within titles and abstracts only (Multimedia Appendix 2).

### Study Selection

The initial screening of titles and abstracts was conducted by the first author [TW]. We used Endnote to detect and delete duplicates, followed by a manual verification by the corresponding author [YMH] to ensure no duplicates were overlooked. After the initial screening of titles and abstracts, a thorough full-text review of selected articles was conducted by 2 reviewers [TW and YMH], adhering to the inclusion and exclusion criteria outlined in Textbox 1. Any discrepancies between the reviewers were resolved through discussion, and if necessary, a third reviewer [HYC] was consulted to reach a consensus. In addition, hand-searching of grey literature was conducted to identify further relevant papers during the full-text review process.

**Inclusion and exclusion criteria of the selected studies.**

**Inclusion criteria**

- Focusing on Asian adults (18 years or older) with at least 1 chronic disease (defined as conditions lasting 1 year or more, requiring ongoing medical attention or limiting activities of daily living or both).
- Using mobile apps to assist patients in self-management and improve patients’ medication adherence.
- Providing descriptions of the features of the apps used in the study.
- Using an experimental design.
- Published in the recent 5 years.

**Exclusion criteria**

- A review article rather than primary research.
- Publications in languages other than English.
- Insufficient detailed information about the mobile app features used in the intervention.
- Not concerning the link between medication adherence and mobile apps.
- Focusing on diseases for which patients do not require long-term medications.

### Data Extraction

Two reviewers [TW and YMH] performed the data extraction process. We jointly decided to extract data from the full-text articles using a pre-established form, which was developed based on literature reviews [10,19,28]. The form encompassed study characteristics, including author, country, and year of publication, as well as details regarding the developer of the mobile app, target population, features, usability, and use of the mobile app. In addition, we documented any additional information about app features not covered in our predefined form. These features of mobile apps across the studies would subsequently be evaluated and compared in our review.

### Data Analysis

The findings are depicted in a narrative table (Table 1), evaluating which apps exhibited the most optimal features using a modified mobile app rating scale derived from literature reviews [10,19,28]. A 10-item evaluation sheet was used to determine the number of features captured by each selected app, with a score assigned based on the total number of items covered (Table 1). A higher score indicated that the mobile app possessed more functions to enhance medication adherence. We summarized and juxtaposed the apps from the selected studies based on their respective disease focus, developers, target users, features, usability, and use. Consequently, we compared and delineated the similarities and disparities in the features of these apps, proposing the ideal features of available apps for enhancing medication adherence.

**Table 1:** Summary and comparison of the selected mobile apps for improving medication adherence.

Study	Disease focus	Developer type	Target user	Features	Number of app functions
Liu et al [29]	Cardiovascular diseases	Research	Health care professional and patient	Education Lifestyle modification (eg, diet, exercise) Reminder (eg, medication-taking, follow-up, and daily monitoring) Others	4
Fan et al [30]	HIV	Research	Health care professional and patient	Education Feedback from health care professionals Motivation Web-based consultation with health care professionals Reminder (eg, medication-taking, follow-up, and daily monitoring) Others	6
Chen et al [31]	Cardiovascular diseases	Research	Caregiver, health care professional, and patient	Data record and monitoring Data export and sharing Education Feedback from health care professionals Lifestyle modification (eg, diet and exercise) Motivation Web-based consultation with health care professionals Patient community forum Reminder (eg, medication-taking, follow-up, and daily monitoring) Others	10
Sunil Kumar et al [32]	Diabetes	Research	Patient	Data record and monitoring Feedback from health care professionals Lifestyle modification (eg, diet and exercise) Reminder (eg, medication-taking, follow-up, and daily monitoring) Others	5
Bozorgi et al [33]	Cardiovascular diseases	Research	Caregiver, health care professional, and patient	Data record and monitoring Data export and sharing Education Feedback from health care professionals Lifestyle modification (eg, diet and exercise) Motivation Reminder (eg, medication-taking, follow-up, and daily monitoring) Others	8
Poorcheraghi et al [34]	Unspecified chronic diseases	Research	Health care professional and patient	Education Feedback from health care professionals Web-based consultation with health care professionals Reminder (eg, medication-taking, follow-up, and daily monitoring) Others	5
Al-Nawayseh et al [35]	Asthma	Research	Patient	Education Others	2
Nurakysh et al [36]	Cardiovascular diseases	Hospital	Caregiver, health care professional, and patient	Data record and monitoring Education Feedback from health care professionals Lifestyle modification (eg, diet and exercise) Motivation Reminder (eg, medication-taking, follow-up, and daily monitoring) Others	7
Yang et al [37]	Diabetes	Commercial company	Health care professional and patient	Data record and monitoring Data export and sharing Feedback from health care professionals Lifestyle modification (eg, diet and exercise) Motivation Web-based consultation with health care professionals	6
Chew et al [38]	Unspecified chronic diseases	Research	Caregiver and patient	Motivation Web-based consultation with health care professionals Reminder (eg, medication-taking, follow-up, and daily monitoring) Others	4
Park et al [39]	Rheumatoid arthritis	Research	Health care professional and patient	Education Web-based consultation with health care professionals Reminder (eg, medication-taking, follow-up, and daily monitoring)	3
Huang et al [40]	Diabetes	Commercial company	Caregiver and patient	Data record and monitoring Data export and sharing Feedback from health care professionals Reminder (eg, medication-taking, follow-up, and daily monitoring) Others	5
Pang et al [41]	HIV	Research	Health care professional and patient	Data export and sharing Feedback from health care professionals Motivation Reminder (eg, medication-taking, follow-up, and daily monitoring) Others	5
Chen et al [42]	Schizophrenia	Research	Health care professional, patient	Data export and sharing Feedback from health care professionals Motivation Web-based consultation with health care professionals Reminder (eg, medication-taking, follow-up, and daily monitoring) Others	6

## Results

### Overview

**Figure 1:**
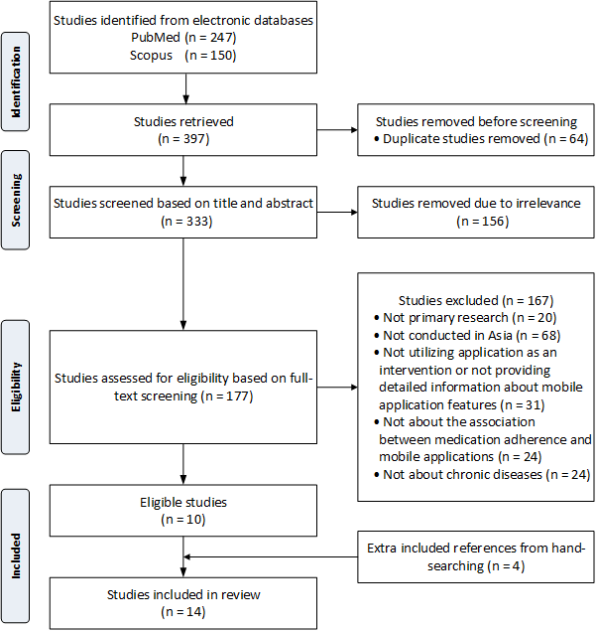
Flowchart of the study selection process.

We initially found 247 articles on PubMed and 150 on Scopus. After removing 64 duplicates, we were left with 333 studies. From this pool, 156 irrelevant articles were excluded based on title and abstract screening. Afterwards, 177 articles underwent full-text review for eligibility assessment. Among these, 167 articles were excluded for various reasons, 20 were not primary research, 68 were not conducted in Asia, 31 did not use mobile apps as an intervention or did not provide detailed information about the mobile apps, 24 did not pertain to the association between medication adherence and mobile apps, and 24 were not relevant to chronic diseases. Ultimately, 14 studies [29-42] were included in our review, with an additional 4 results [32,33,35,39] obtained from hand-searching, as illustrated in Figure 1.

### Characteristics of the Included Studies

The majority of the studies (9/14, 64.3%) were conducted in East and Southeast Asia [29-31,37-42], with the remaining studies conducted in India (1/14, 7.1%) [32], Iran (2/14, 14.3%) [33,34], Jordan (1/14, 7.1%) [35], and Kazakhstan (1/14, 7.1%) [36], respectively. All the studies investigated the use of mobile apps to improve medication adherence among patients with chronic diseases, defined as conditions lasting 1 year or more, requiring ongoing medical attention, or restricting activities of daily living. Among these studies, 4 (28.6%) targeted patients with cardiovascular diseases [29,31,33,36], 3 (21.4%) focused on diabetes mellitus [32,37,40], 2 (14.3%) on HIV management [30,41], 1 (7.1%) on schizophrenia care [42], 1 (7.1%) on asthma treatment [35], 1 (7.1%) on rheumatoid arthritis [39], and 2 (14.3%) included patients with various chronic diseases and polypharmacy [34,38].

### Features of the Mobile Apps

Among the mobile apps in these studies, 11 (78.6%) were developed by research teams [29-35,38,39,41,42], while 3 (21.4%) were existing apps developed by commercial companies or hospitals [36,37,40]. The features of the 14 mobile apps are summarized in Table 1, showing significant differences among them. All of the selected apps incorporated multiple features to improve medication adherence. Reminders were the most commonly used feature, while patient community forums were the least integrated feature in the app design.

In total, 12 (85.7%) apps featured a reminder function primarily aimed at reminding patients to take their medications as scheduled [29-36,38-40,42]. Some of these apps also provided reminders for follow-up appointments [30,31,33,35,40], prescription refills [40], or monitoring health status (eg, body weight, blood pressure, or blood sugar) [31,33,35,40]. Out of 14, 6 (42.9%) apps offered lifestyle modification functions by providing dietary and exercise recommendations and generating individualized plans and monitoring [29,31-33,36,37]. Furthermore, 6 (42.9%) apps included health data recording and monitoring functions (eg, body weight, blood pressure, and blood sugar) [29,31-33,36,37], with 4 (28.6%) allowing users to export and share these records with researchers or health care professionals [29,32,33,37]. Two (14.3%) other apps, while unable to record health data, allowed medication intake records to be uploaded and shared with researchers or health care professionals [31,36]. A higher proportion of the apps we reviewed had communication features with researchers or health care professionals. Specifically, 10 (71.4%) apps enabled users to receive feedback from researchers or health care professionals during their usage [30-37,40,42], and 7 (50.0%) apps offered web-based consultation services [30,31,34,37-39,42]. Furthermore, some apps took measures to reinforce patients’ willingness to adhere to medications. Eight (57.1%) apps included educational content for patients [29-31,33-36,39], while 7 (50.0%) used motivation strategies to encourage adherence [31,33,35-38,42]. Only 1 app (7.1%) offered a patient community forum, allowing patients to share and discuss their experiences with medication and disease management with peers and caregivers in a safe space [31].

Several unique features were identified across the selected apps in this review (Multimedia Appendix 3). HeartGuardian (Liu et al) supported the management of lipid levels and improved medication adherence by tailoring detailed health plans for patients, recording medication intake, and providing real-time video feedback based on their adherence [29]. Medisafe app offered an easy-to-use reminder feature to help patients take their medications on time while also tracking blood pressure or blood sugar levels. It also assessed patients’ health data and medication adherence and allowed caregivers to supervise medication intake [40]. Pang et al [41] created the Mobile Interactive Supervised Therapy (MIST) system to monitor medication adherence among people with HIV, requiring patients to upload videos of their medication intake for researchers to review. Chew et al [38] developed the Med Assist app, which included a 2-way alarm reminder system, allowing users to take medications later if they miss the scheduled time. It could also summarize all medications that patients had to take into a single page and help caregivers supervise medication usage. Furthermore, patients could use the Med Assist app to check medication availability at partnered pharmacies, making prescription refills more convenient. The Med Assist app also featured an adherence scoring system, incorporating gamification where users earned rewards for achieving app objectives [38]. DIAGURU, a mobile app created by Sunil Kumar et al for diabetes management, was capable of recording and monitoring patients’ blood sugar levels, insulin usage, and food intake to generate visualized reports and graphs as feedback [32]. In addition, DIAGURU could calculate the caloric value of food intake and provide dietary recommendations based on patients’ sugar levels and the caloric consumption [32]. Poorcheraghi et al [34] designed a medication management app targeting older adults, featuring user-friendliness, adjustable font and text sizes, and appropriate background and app item colors. The app recorded the names and images of medications and would audibly announce the medication names and show their pictures when playing the reminders [34]. Asthma mHealth, developed by Al-Nawayseh et al [35], focused on educational functions, providing patients with detailed introduction about asthma and using short videos to demonstrate correct inhaler usage.

Finally, only 5 studies (35.7%) included descriptions of privacy protection measures [30,31,34,39,40]. The most detailed information was provided by Chen et al [31], who implemented user authentication, data encryption for both transmission and storage, and strong password protection for the iCARE app. In addition, the iCARE featured backup and recovery functions and used MongoDB (one of open-source NoSQL databases) for its flexibility and security [31]. The app’s development also adhered to international and national data standards [31].

### Usability and Use of the Selected Mobile Apps

Although the majority of studies in our review were still in the early stages of app development and pilot testing, 12 (85.7%) reported improvements in clinical or humanistic outcomes [29,31-38,40-42]. As summarized in Table 2, these findings underscore the potential effectiveness of mobile apps in enhancing patient outcomes from clinical and humanistic perspectives.

**Table 2:** Outcome measures of the selected mobile apps for improving medication adherence.

Study	Clinical outcomes	Humanistic outcomes
Liu et al [29]	Blood lipid Medication-associated adverse events	Medication adherence
Fan et al [30]^a^	—^b^	—^b^
Chen et al [31]	—^b^	Satisfaction Usefulness
Sunil Kumar et al [32]	—^b^	Quality of life
Bozorgi et al [33]	Blood pressure Physical activity	Medication adherence Satisfaction Usefulness
Poorcheraghi et al [34]	Medication-associated adverse events	Medication adherence
Al-Nawayseh et al [35]	Disease status	Ease of use Medication adherence Satisfaction Usefulness
Nurakysh et al [36]	—^b^	Medication adherence
Yang et al [37]	Blood glucose Blood pressure	Medication adherence Satisfaction
Chew et al [38]	—^b^	Ease of use Satisfaction Usefulness
Park et al [39]^a^	—^b^	—^b^
Huang et al [40]	—^b^	Ease of use Medication adherence Satisfaction Usefulness
Pang et al [41]	Disease status	Ease of use Usefulness
Chen et al [42]	—^b^	Medication adherence

^a^The study protocol did not include findings on the effectiveness of apps in terms of patient outcomes.

^b^Not applicable.

Six (42.9%) studies highlighted that mobile apps lead to clinical improvement [29,33-35,37,42], including blood glucose, blood pressure, blood lipids, disease status, reduction in medication associated adverse events, and increased physical activities. In addition, 12 (85.7%) studies reported positive humanistic outcomes such as increased medication adherence, improved quality of life, and positive attitude toward the mobile apps, including satisfaction, usability, and ease of use [29,31-38,40-42]. Among these, 8 (57.1%) studies noted better medication adherence in intervention groups using mobile apps compared with control groups [29,33-37,40,42]. Furthermore, 7 (50.0%) studies included in our review assessed users’ attitudes toward the mobile apps [31,33,35,37,38,40,41]. Of these, 6 (42.9%) reported positive user attitude and satisfaction with the apps [31,33,35,37,38,40], 6 (42.9%) considered the apps useful and beneficial [31,33,35,38,40,41], and 4 (28.6%) found the apps easy to use [35,38,40,41]. One (7.1%) study suggested that mobile apps could serve as a public health tool to enhance patients’ quality of life [32].

Some studies also provided user feedback or detailed how the mobile apps were used. For instance, Chen et al [31] noted that the most frequently visited screens were those related to health recommendations, daily monitoring of health indicators, health behaviors, and medication adherence. Chew et al [38] found that features such as reminder functions, medication summary, and multiuser support were particularly helpful, though users faced challenges when adding new medications and suggested more in-app guidance. Huang et al [40] observed that while the app was useful for older users, they found reminder notifications somewhat annoying. Finally, Pang et al [41] reported that users appreciated features such as medication reminders, a color-coded calendar, and a reward system, though there were privacy concerns about features that might inadvertently disclose users’ HIV status.

## Discussion

### Principal Findings

This narrative review identified 14 mobile apps used to enhance medication adherence within the Asian context. The findings illustrate the varied features of existing mobile apps used to address medication adherence, aiming for improved health outcomes. Consistent with previous research on enhancing medication adherence, integrating a variety of reminder, behavioral, and educational strategies into intervention content has potential to enhance adherence behavior more efficiently and effectively [9].

Using reminder strategies is a key tactic to help patients adhere to their medication schedules, often implemented through alarms, push notifications, or short message services [19]. To ensure correct medication use, patients need to input or log their medication information into apps, such as the name, dosage, and frequency of their medications. This process requires a level of proficiency in using digital devices. Patients may benefit from user-friendly interfaces or additional assistance from family members or health care professionals to navigate these apps effectively. Furthermore, some apps remind patients of regular follow-up appointments and disease monitoring, providing additional cues to reinforce patient self-efficacy and mitigate forgetfulness, which is one of the most commonly reported reasons for nonadherence to chronic disease medications [4,43]. Therefore, integrating reminder functions for medication-taking with other aspects of disease management may result in improved medication adherence [34,41].

Behavior-focused strategies, such as prompts, social support, and rewards, are effective in encouraging patients to adopt new medication practices [9]. A higher proportion of feedback and web-based consultation features were observed in the mobile apps reviewed in this study. In contrast, many medication adherence apps available on the Apple App Store or Google Play Store lack such functions [10,28]. This divergence may stem from the fact that the apps in our review were mostly developed by research teams for study purposes. This enabled them to integrate more health care professional support for feedback and web-based consultation features. However, most mobile apps available to patients on their personal smartphones are patient-focused and not accessible by pharmacists or physicians [10]. Integrating chatbot functionality to facilitate mutual conversation between patients and health care professionals to address patient concerns requires costly infrastructure, making it more common in research settings than for commercial purposes. Further research is needed to confirm the effectiveness of these features.

In addition, several motivation systems were identified in the apps to motivate patients to adhere to medication regimens. Two apps provided messages about praise and encouragement for patients with good adherence [33,37], while 5 apps used gamification or financial incentives as motivation strategies [31,35,38,41,42]. Patients would receive rewards such as digital coins, scenic photos, or local currency upon confirming their medication adherence. Consistent with previous studies, our review indicated that reward and gamification-based medication adherence features could effectively improve or maintain optimal medication adherence [44,45]. However, the precise correlation and ceiling effect of the incentive value and its impact on medication adherence required further investigation.

Socialization features are valuable for patients as they offer social support from family, friends, peers, and health care professionals [46]. Patient community forums create comfort space for patients with similar backgrounds to share their experiences of tackling medications, enabling immediate consultation with health care professionals or fellow patients in case of any medication-related concerns [47]. Among the apps reviewed, only the iCARE included such a feature. Moving forward, the development of medication adherence apps may consider incorporating such a socialization feature [47].

Apart from medication intake, disease management relies on dietary adjustments and lifestyle modifications [5]. The educational strategies used by the selected apps encompassed various aspects, including disease background, medication introduction, diet guidance, and exercise recommendations. While it is recognized that simply providing knowledge may not always lead to behavior change, offering basic information can assist patients in navigating their health care journey, preventing the consolidation of misinformation. This is particularly beneficial for individuals facing barriers to accessing credible information for medication use and disease management.

A team-based health care approach facilitates collaboration among patients, caregivers, and providers, enhancing health care satisfaction and patient outcomes [30,31]. Implementing the multiple user support function enables caregivers, such as family members, to remotely monitor patients’ medication adherence and health status, taking appropriate actions when necessary [37]. However, in our review, only 3 mobile apps included such functionality [31,33,36], grouping patients, caregivers, and health care professionals as target users to enhance medication adherence. Other apps primarily focused on delivering information to patients themselves [32,35] or facilitating communication between patients and their health care providers [29,30,34,37,41,42], overlooking the role of caregivers. Caregivers play a crucial role in supporting patients with chronic diseases and have been shown to enhance patients’ medication adherence and treatment outcomes [40]. Participants in the study by Chew et al [37] also expressed high satisfaction with the multiple user support function of the Med Assist, particularly as it is common for working adults to care for older parents in the Asian community, making multiple-user support a convenient and effective method. In summary, the inclusion of multiple user support functionality may be considered an important feature in future mobile app development, provided privacy concerns are addressed.

Privacy and confidentiality concerns can significantly impact the acceptability of mobile apps [41]. Given that these apps often store sensitive data, such as personal identification information, ensuring system security and protecting user privacy is crucial [39]. Measures such as user authentication, encryption for data transmission and storage, and strong password protection are essential for safeguarding privacy [30,31,34,39,40]. In addition, app features should be carefully designed to prevent unintended disclosure of users’ medical conditions. For instance, while the MIST app requires patients to record and report videos of their medication intake, some users worry that this could inadvertently reveal their HIV status, as it might attract unwanted attention from others [41]. Although this feature may improve medication adherence, it also raises concerns about privacy. Currently, there are no unified regulations or guidelines for privacy protection in mobile health apps, making it important for future developments to prioritize privacy safeguards to maintain user trust and protect sensitive information [36].

None of the selected apps were developed by the government, potentially limiting their use in connecting patient health information across institutions. In some Asian countries with single-payer public health insurance, integrating patient data through a single-payer health care system is more feasible than dealing with data fragmentation from a multipayer health care system [48]. The majority of the mobile apps reviewed were developed by research teams, while others were developed by commercial companies or hospitals. None of the selected apps were able to synch to other adherence devices. Consequently, patient health information recorded in these mobile apps may only be accessible to a few health care networks collaborating with the app developers and may not be easily assessed or updated within other health care systems without special requests or permissions. Incorporating patient-generated health data, which includes clinically relevant information captured by patients outside of conventional health care settings, into electronic health records is essential. This integration is critical because leveraging patient-generated health data in clinical practice has the potential to fill health care gaps and enhance personalized treatment [49]. Furthermore, integrating health services into a single reliable and personalized platform could streamline the health care journey, potentially incentivizing patients to adhere to using a single app [40]. While consolidating patient information from various sources into 1 platform can simplify health care processes, developers need to prioritize data security and personal confidentiality. This is essential to prevent users from avoiding or uninstalling an app due to concerns about privacy breaches [12].

Although the mobile apps reviewed in this study come from various Asian countries with differing design features and cultural contexts, we found consistently high user satisfaction and perceived usefulness [31,33,35,37,38,40,41]. Most studies also reported that these apps positively impacted clinical outcomes and medication adherence [29,33-37,40,42]. These results align with previous research conducted in western populations [10,50-52], indicating that mobile app interventions for patients with chronic diseases are both feasible and well-received in the Asian population as well.

Overall, the selected mobile apps consist of a broad spectrum of components to address medication adherence. While these apps offer diverse features for improving medication adherence, it is important to note that the quality and effectiveness of an app cannot be solely determined by the number of features it provides. Without valid studies, it remains unclear which apps are most suitable for use. Recent evidence suggests a shift in the trend toward the use of digital devices as interest in mHealth grows [53]. Therefore, developers should consider users’ needs, their proficiency with digital devices, and the resources available to them when designing apps to promote medication adherence.

### Limitations of the Research

This study has several limitations. First, the apps selected were obtained from peer-reviewed journals and not from commercial platforms such as the Apple App Store and Google Play Store. Therefore, the included mobile apps may not represent all available options, particularly those not published in academic databases. It is worth noting that paid commercial apps may offer additional features and functionality, and testing such apps could have provided further insights into strategies used to promote medication adherence. Second, we only included articles available in English, potentially overlooking effective apps in other languages. Third, our search was limited to articles published until August 2024, and given the rapid production and release of new apps, we acknowledge that new adherence apps may have been released after our review.

### Conclusion

This review has highlighted key components integrated into mobile apps for medication adherence. Nevertheless, the lack of government and corporate engagement during development impedes the generalizability of any single app. In addition, beyond basic reminder functions, features such as multiple user support, feedback mechanisms, web-based consultation, motivational tools, and socialization features show promising potential in improving medication adherence. Further pragmatic research is essential to confirm the efficacy of these selected apps in enhancing medication adherence.

### Data Availability

The study materials and detailed analyses are available from the corresponding author upon reasonable request.

## Appendix

Multimedia Appendix 1 Using the SPIDER (sample, phenomenon of interest, design, evaluation, research type) framework to create the research question.

Multimedia Appendix 2 Searching process on PubMed and Scopus.

Multimedia Appendix 3 Unique features of the selected mobile apps for improving medication adherence.

## Abbreviations

MIST: Mobile Interactive Supervised Therapy
SPIDER: sample, phenomenon of interest, design, evaluation, research type
