# Supplementary material for: Exploration of Features of Mobile Applications for Medication Adherence in Asia: Narrative Review
Source: J Med Internet Res. 2024 Nov 8;26:e60787. doi: 10.2196/60787 (PMC11584533; doi:10.2196/60787)
Supplement: Multimedia Appendix 1 [file jmir_v26i1e60787_app1.docx]

**Appendix 1.** Using the SPIDER framework to create the research question

| SPIDER tool | Definition | Search Terms/Strategies |
| --- | --- | --- |
| S - Sample | Asian countries, chronic disease | “Asia” OR “chronic disease” |
| PI - Phenomenon of Interest | features of mobile applications | “app” OR “application” |
| D - Design | intervention | “survey” OR “experiment” OR “questionnaire” OR “group” |
| E - Evaluation | medication adherence | “medical adherence” OR “medication adherence” |
| R - Research Type | qualitative, quantitative, or mixed methods | “case control” OR “cohort study” OR “randomized controlled trial” OR “clinical trial” OR “observational study” OR “qualitative research” OR “mixed methods” OR “analysis” |
