# Supplementary material for: Exploration of Features of Mobile Applications for Medication Adherence in Asia: Narrative Review
Source: J Med Internet Res. 2024 Nov 8;26:e60787. doi: 10.2196/60787 (PMC11584533; doi:10.2196/60787)
Supplement: Multimedia Appendix 2 [file jmir_v26i1e60787_app2.docx]

**Appendix 2.** Searching process on PubMed and Scopus

### Search Term for PubMed

(((Asia) OR (Chronic disease)) AND ((App) OR (Application))) AND ((((Survey) OR (Experiment) OR (Questionnaire) OR (Group)) AND ((Medication adherence) OR (Medical adherence))) AND ((Case Control) OR (Cohort Study) OR (Randomized Controlled Trial) OR (Clinical Trial) OR (Observational Study) OR (Qualitative Research) OR (Mixed Methods) OR (Analysis)))

Filters: Abstract, in the last 5 years / Sort by: Most Recent

### Search Term for Scopus

TITLE-ABS-KEY(( ( ( asia ) OR ( chronic AND disease ) ) AND ( ( app ) OR ( application ) ) ) AND ( ( ( ( survey ) OR ( experiment ) OR ( questionnaire ) OR ( group ) ) AND ( ( medication AND adherence ) OR ( medical AND adherence ) ) ) AND ( ( case AND control ) OR ( cohort AND study ) OR ( randomized AND controlled AND trial ) OR ( clinical AND trial ) OR ( observational AND study ) OR ( qualitative AND research ) OR ( mixed AND methods ) OR ( analysis ) ) )) AND PUBYEAR > 2018 AND PUBYEAR < 2025
