# Supplementary material for: Exploration of Features of Mobile Applications for Medication Adherence in Asia: Narrative Review
Source: J Med Internet Res. 2024 Nov 8;26:e60787. doi: 10.2196/60787 (PMC11584533; doi:10.2196/60787)
Supplement: Multimedia Appendix 3 [file jmir_v26i1e60787_app3.docx]

**Appendix 3.** Unique features of the selected mobile apps for improving medication adherence.

| **Study** | **Name of the application** | **Special features** |
| --- | --- | --- |
| Liu et al., China, 2019 [29] | HeartGuardian | • Health education including tailored health plan and daily updates on lifestyle, diet, and treatment.  • Real-time video feedback for self-empowerment based on medication adherence. |
| Fan et al., China, 2020 [30] | - | • Linked to WeChat (a social media platform in China) for convenient communication with case managers. |
| Chen et al., China, 2021 [31] | iCARE | • Provide medication recommendations through visualization techniques (graphical or image-based format).  • Establish personalized risk management goals based on the patients’ initial health information and could modify the goals according to feedback from the patients, caregivers, and healthcare professionals. |
| Sunil Kumar et al., India, 2020 [32] | DIAGURU | • Record and monitor patients’ blood sugar, insulin usage, and food intake to generate visualized reports and graphs as feedback. |
| Bozorgi et al., Iran, 2021 [33] | BPMAP | • Allow caregivers to access the application and notify them of critical blood pressure levels.  • Send general motivational messages and individualized messages based on patient characteristics. |
| Poorcheraghi et al., Iran, 2023 [34] | - | • Feature user-friendliness with adjustable font sizes, text sizes, and suitable colors for the elderly.  • Record medication names and images, audibly announce names, and show pictures during reminders. |
| Al-Nawayseh et al., Jordan, 2021 [35] | Asthma mHealth | • Provide patients with detailed introduction about asthma and using short videos to demonstrate correct inhaler usage. |
| Nurakysh et al., Kazakhstan, 2022 [36] | MyTherapy | • Remind patients of medication refills.  • Generate monthly PDF reports for discussions with healthcare professionals. |
| Chew et al., Malaysia, 2020 [38] | Med Assist | • Two-way alarm reminder system.  • Summarize all medications patients need to take on one page.  • Assist in checking medication availability at partnered pharmacies. |
| Huang et al., Singapore, 2019 [40] | Medisafe | • Assessment of patients’ health data and medication adherence and enable caregivers’ supervision through “Medfriend” function. |
| Pang et al., Singapore, 2020 [41] | MIST | • Require patients to upload videos of medication intake for researchers review. |
| Chen et al., Taiwan, 2023 [42] | MedAdhere | • Identify the patient’s face and antipsychotic medications’ appearance through the mobile phone camera  • Linked to Line (a social application in Taiwan) for convenient communication with case managers. |
